# Brain 5-HT Deficiency Prevents Antidepressant-Like Effects of High-Fat-Diet and Blocks High-Fat-Diet-Induced GSK3β Phosphorylation in the Hippocampus

**DOI:** 10.3389/fnmol.2019.00298

**Published:** 2019-12-11

**Authors:** Michelle M. Karth, Brittany J. Baugher, Nicole Daly, Melinda D. Karth, Stephen C. Gironda, Benjamin D. Sachs

**Affiliations:** Department of Psychological and Brain Sciences, College of Liberal Arts and Sciences, Villanova University, Villanova, PA, United States

**Keywords:** serotonin, depression, anxiety, mouse model, high fat and calories diet

## Abstract

Obesity is associated with an increased risk of depression and anxiety disorders, but the nature of the relationship(s) between obesity and mental illness remains highly controversial. Some argue that depression and anxiety lead to increased consumption of “comfort foods,” the intake of which reduces negative affect and promotes obesity. In contrast, others have theorized that negative affect results from chronic excessive consumption of highly palatable foods. The brain serotonin (5-HT) system has long been implicated in both the development and treatment of mental illness. Preclinical studies have shown that low brain 5-HT exacerbates depression- and anxiety-like behaviors induced by stress and blocks reductions in depression-like behavior induced by antidepressants, but the effects of brain 5-HT deficiency on responses to high-fat diet (HFD) have not been explored. The current work used genetically modified mice to evaluate the effects of low 5-HT on behavioral and molecular alterations induced by chronic exposure to HFD. Our results reveal that HFD decreases depression-like behavior and increases some anxiety-like behaviors in wild-type (WT) mice. However, genetic brain 5-HT deficiency blocks HFD-induced reductions in forced swim immobility and prevents HFD-induced increases in hippocampal GSK3β phosphorylation despite having no significant effects on HFD-induced changes in body weight or anxiety-like behavior. Together, our results suggest that brain 5-HT deficiency significantly impacts a subset of behavioral and molecular responses to HFD, a finding that could help explain the complex relationships between obesity and mental illness.

## Introduction

Major depressive disorder is currently ranked by the Global Burden of Disease Report as the second leading cause of worldwide disability (Ferrari et al., 2014), and anxiety disorders are debilitating conditions that affect approximately 20% of the US population (Kessler et al., 2012). Although there are numerous factors that likely contribute to the development of these mental illnesses, accumulating data indicate that obesity can increase the risk of both depression (Luppino et al., 2010; Li et al., 2017) and anxiety disorders (Gariepy et al., 2010). Despite the high degree of comorbidity between obesity and affective disorders (Faith et al., 2002; Stunkard et al., 2003), the effects of obesity on depression- and anxiety-like behaviors remain debated. Several recent preclinical studies have reported that high-fat diet (HFD) can increase anxiety- and depression-like behaviors across a range of species, including nonhuman primates (Sullivan et al., 2010), mice (Sharma and Fulton, 2013; Strekalova et al., 2015; Almeida-Suhett et al., 2017; Bridgewater et al., 2017; Kurhe et al., 2017; Wu et al., 2018; Xu et al., 2018; Ogrodnik et al., 2019), and rats (Abildgaard et al., 2011; Aslani et al., 2015; Dutheil et al., 2016; Rebolledo-Solleiro et al., 2017; Alonso-Caraballo et al., 2019). However, multiple other reports have noted *reductions* in depression- and/or anxiety-like behavior following chronic consumption of HFD (Maniam and Morris, 2010a,b; Finger et al., 2011; Dornellas et al., 2018). Although the reasons for these discrepant findings are currently unknown, it is likely that genetic factors could influence behavioral responses to HFD. To evaluate the impact of genetically induced brain 5-HT deficiency on changes in body weight and depression- and anxiety-like behaviors following chronic HFD, the current work examined the tryptophan hydroxylase 2 (Tph2) R439H knock-in (KI) mouse line, which harbors a partial loss-of-function mutation in the brain 5-HT synthesis enzyme, Tph2 (Beaulieu et al., 2008). Homozygous KI animals from this line have 60–80% less brain 5-HT than their homozygous wild-type (WT) littermates (Beaulieu et al., 2008; Jacobsen et al., 2012). These animals have been shown to exhibit increased susceptibility to anxiety- and depression-like behavior induced by stress (Sachs et al., 2015), but whether low levels of brain 5-HT alter behavioral responses to other potential environmental risk factors for mental illness (such as HFD) has not been established.

The mechanisms through which HFD might influence depression- and anxiety-like behaviors are not completely understood, but preclinical work has suggested a potential role of HFD-induced alterations in GSK3β signaling (Papazoglou et al., 2015; Wakabayashi and Kunugi, 2019) and brain inflammation (Dutheil et al., 2016; Wu et al., 2018). In particular, the upregulation of several pro-inflammatory cytokines in the brain, including interleukin-1β (IL-1β; Almeida-Suhett et al., 2017) and interleukin-6 (IL-6; Wakabayashi and Kunugi, 2019), has been implicated in murine behavioral responses to HFD. Dysregulation of GSK3β (Jope, 2011; Karege et al., 2012; Ren et al., 2013; Ronai et al., 2014; Chen et al., 2015) and inflammation (Syed et al., 2018; Giridharan et al., 2019; Opel et al., 2019; Osimo et al., 2019) have both been identified in clinical studies examining psychiatric patients as well, thus supporting their likely importance in behavioral dysfunction. Given that both brain inflammation (Lu et al., 2017; Khodanovich et al., 2018) and GSK3β activity (Li et al., 2004; Beaulieu et al., 2008) are known to be influenced by brain 5-HT levels, the current work examined whether low 5-HT impacts the effects of HFD on GSK3β phosphorylation or the mRNA expression of several genes involved in inflammation. Although 5-HT could influence HFD responses through both peripheral and central mechanisms, the use of Tph2KI mice limits the present study’s focus on central mechanisms. Although the inhibition of peripheral 5-HT synthesis has been shown to lead to resistance to HFD-induced obesity (Crane et al., 2015) and can attenuate HFD-induced depression-like behavior (Pan et al., 2019), the current study is the first to evaluate the impact of genetically induced brain 5-HT deficiency on behavioral and molecular responses to HFD.

## Method

### Animals

The male homozygous WT and homozygous KI animals from the Tph2R439H mouse line used for this study were generated *via* heterozygous breeding at Villanova University. This line has been backcrossed to the C57BL/6 line for 10 generations, and littermates were used as controls. Adult mice were used for all experiments, and HFD exposure began when mice were 2–4 months of age. There were no differences in the average age of mice in any of the treatment groups. All studies were performed in accordance with protocols that were approved by the Institutional Animal Care and Use Committee (IACUC).

### Diet and Housing

All mice were fed Envigo’s Teklad Global Diet (standard natural ingredient diet: ID #2019, 19% protein, 9% fat, and 3.3 kcal/g) from the time of weaning until the start of HFD exposure. The HFD groups were fed Envigo’s Teklad Custom Diet (adjusted fat diet: ID #95217, 18.8% protein, 39.7% fat, and 4.3 kcal/g) for a total of 22 weeks, whereas the standard diet (SD) groups continued receiving Envigo’s #2019 diet throughout the study. Food and water were available *ad libitum*, and the animals were housed in a temperature- and humidity-controlled room. Mice were group housed (three to five per cage), maintained on a 12-h light-dark cycle, and weighed weekly throughout the course of the experiment. Behavioral testing began after the mice had been on HFD (or SD) for 20 weeks. The order of testing was as follows: open field test (OFT), week 20, Friday; elevated plus maze (EPM), week 21, Monday; and forced swim test (FST), week 21, Friday. Behavioral testing was conducted during the light phase of the light-dark cycle, typically starting ~5 h prior to the start of the dark phase.

Separate cohorts of WT and KI mice were singly housed to allow for measurements of food intake. Twenty-four-hour consumption of SD and HFD was measured once weekly.

### Open Field Test

For the OFT, exploration and locomotor activity were assessed in 40 cm × 40 cm plexiglass ANY-box activity chambers (Stoelting, Wood Dale, IL, USA), with a center square measurement of 20 cm × 20 cm. Each mouse was placed into a corner of the open field, and its movement and location were recorded for 20 min with overhead digital cameras. The total distance traveled, the distance traveled in the center, the number of entries to the center, and the time spent in the center were all calculated using ANY-maze software.

### Elevated Plus Maze

For the EPM, the location and activity of mice in the apparatus were measured using ANY-maze animal tracking software. The time spent and distance traveled in the open arms, the closed arms, and entire apparatus were determined for each mouse.

### Forced Swim Test

The FST was performed essentially as we have previously reported (Sachs et al., 2014a). Briefly, mice were placed in a 4-L beaker filled with approximately 2,500 ml of 25°C water, where they remained for 6 min. The behavior of each mouse was recorded from above using a camera suspended above the beaker. ANY-maze software was used to measure the amount of time that each mouse spent immobile and the number of immobile episodes.

### Gene Expression Analysis

One week following the FST, mice were killed by cervical dislocation and decapitation, after which the brains were removed and sectioned into 1-mm-thick sections using a brain matrix. Bilateral tissue punches (1.5 mm in diameter and 1 mm in thickness) were taken from the hippocampus [centered approximately ±1.0 mm medio-lateral (ML), −2.0 mm anterior–posterior (AP), −1.7 mm dorso-ventral (DV) relative to Bregma] and immediately frozen on dry ice and transferred to a −70°C freezer until further processing. RNA was isolated from the tissue samples collected from the hippocampus of the left hemisphere using the Ambion PureLink RNA Mini Kit, according to the manufacturer’s instructions. RNA was frozen at −70°C until further processing.

Reverse transcriptions were performed using the Thermo Scientific Maxima First Strand cDNA Synthesis Kit, according to the manufacturer’s instructions. Real-time polymerase chain reaction (RT-PCR) was performed as we have described previously (Sachs et al., 2018) using the PowerUp Sybr Green Master Mix rt-PCR kit (Applied Biosystems, Foster City, CA, USA) according to the manufacturer’s instructions. Primer sequences were selected from PrimerBank (Wang et al., 2012): GAPDH forward: 5′-CATGTTCCAGTATGACTCCACTC-3′; GAPDH reverse: 5′-GGCCTCACCCCATTTGATGT-3′; complement C4A forward: 5′-GATGACAAGAACGTGAGTGTCC-3′; complement C4A reverse: 5′-CCCTTTAGCCACCAATTTCAGG-3′; IL-1β forward: 5′-TTCAGGCAGGCAGTATCACTC-3′; IL-1β reverse: 5′-GAAGGTCCACGGGAAAGACAC-3′; IL-6 forward: 5′-TCTATACCACTTCACAAGTCGGA-3′; IL-6 reverse: 5′-GAATTGCCATTGCACAACTCTTT-3′; ionized calcium binding adaptor molecule 1 (IBA1) forward: 5′-ATCAACAAGCAATTCCTCGATGA-3′; IBA1 reverse: 5′-CAGCATTCGCTTCAAGGACATA-3′; GSK3β forward: 5′-ACAGGCCACAGGAGGTCAGT-3′; GSK3β reverse: 5′-GATGGCAACCAGTTCTCCAG-3′.

### Western Blotting

Hippocampal tissue punches from the right hemisphere were processed for Western blotting essentially as we have described previously (Sachs et al., 2014b). Briefly, samples were lysed in ice-cold lysis buffer (1% Triton, 1 mM of EDTA, 150 mM of NaCl, and 20 mM of Tris–HCl) with protease and phosphatase inhibitors added. A small aliquot was used for protein determinations using bicinchoninic acid (BCA) assay kits (Boston Bio Products, Ashland, MA, USA). Equal amounts of protein were loaded onto TGX gels for electrophoresis. Proteins were then transferred to polyvinylidene difluoride (PVDF) membranes, and Western blotting was performed using the following antibodies: rabbit anti-phosphorylated GSK3β (Cell Signaling, #9323, 1:300 dilution), mouse anti-total GSK3β (Cell Signaling, #9832, 1:300 dilution), and rabbit-anti-GAPDH as a loading control (Cell Signaling, #5174, 1:300 dilution).

### Statistical Analysis

For weight gain, SPSS software was used to conduct a three-factor ANOVA with two between-subjects factors (i.e., genotype and diet) and one within-subjects repeated-measures factor (i.e., time). All other data were analyzed by two-way ANOVA with genotype and diet as factors using JMP software.

## Results

### High-Fat-Diet-Induced Weight Gain

**Figure 1:**
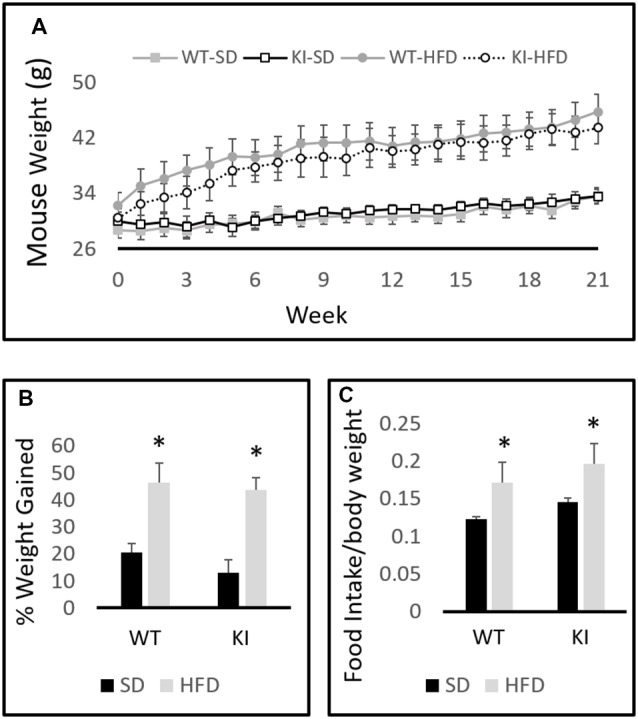
Body weights of wild-type (WT) and knock-in (KI) mice on standard diet (SD) and high-fat diet (HFD). **(A)** Quantification of average body weights for each group over time. **(B)** Calculation of average percent body weight gain over the 21-weeks period for each group. **(C)** Average daily food intake in mice on SD or HFD. Results are expressed as the mean, and error bars indicate standard error. “*” Indicates main effect of diet by two-way ANOVA, *p* < 0.05. *N* = 6–11 per group.

A between-subjects two-by-two ANOVA with diet and genotype as factors revealed a significant effect of diet on weight (*F*_(1,30)_ = 19.6, *p* < 0.0001, Figure 1A) but no effect of genotype (*F*_(1,30)_ = 0.001, *p* = 0.97, Figure 1A). A within-subjects repeated-measures analysis revealed a significant effect of time on body weight (*F*_(21,10)_ = 69.2, *p* < 0.0001, Figure 1A) and a significant time by diet interaction (*F*_(21,10)_ = 15.0, *p* < 0.0001, Figure 1A). Chronic exposure to HFD led to a significantly greater increase in body weight in both WT and KI animals than did continued exposure to SD, but no significant genotype differences in body weight were observed at any time point (Figure 1A). As expected, animals treated with HFD increased their weights by a significantly higher percentage than SD-fed mice over the course of the experiment (*F*_(1,30)_ = 34.98, *p* < 0.0001, Figure 1B). Comparisons of 24-h food intake in WT and KI mice consuming either SD or HFD revealed no significant genotype differences in consumption, but mice ate significantly more HFD per body weight compared with SD (main effect of diet by two-way ANOVA, *F*_(1,24)_ = 5.88, *p* = 0.023, Figure 1C).

### Open Field Test

**Figure 2:**
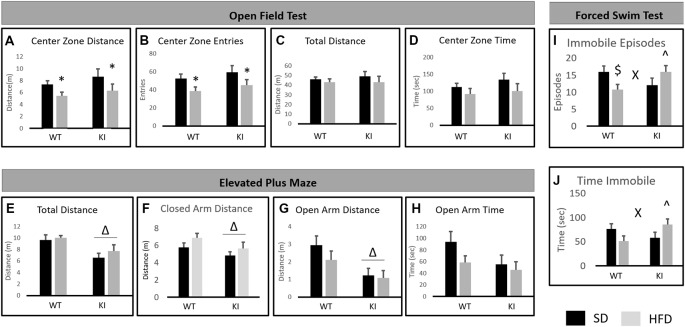
Behavioral consequences of chronic HFD on WT and KI mice. **(A)** The average distance traveled in the center of the open field for each group. **(B)** The average number of entries into the center of the open field for each group. **(C)** The average distance traveled in the entire open field for each group. **(D)** The time spent in the center of the open field in each group. **(E)** Total distance traveled in the elevated plus maze (EPM). **(F)** Distance traveled in the closed arms. **(G)** Distance traveled in the open arms. **(H)** Time spent in the open arms of the plus maze. **(I)** The number of immobile episodes in the forced swim test (FST). **(J)** The time spent immobile in the FST. Results are expressed as the mean, and error bars indicate standard error of the mean. “Δ” Indicates a main effect of genotype, “*” indicates a main effect of diet, and “X” indicates a significant genotype by diet interaction by two-way ANOVA, *p* < 0.05. “∧” Indicates significant increase compared with WT-HFD, and “$” indicates significant decrease compared to WT-SD by *post hoc* *t*-tests. *N* = 10–11 per group.

For the OFT, there was a main effect of diet, with HFD decreasing the distance traveled in the center of the field (main effect of diet; *F*_(1,35)_ = 5.58, *p* = 0.024, Figure 2A) and decreasing the number of entries made to the center of the field (main effect of diet; *F*_(1,35)_ = 6.29, *p* = 0.019, Figure 2B). However, there was no significant overall effect of diet on locomotor activity (*F*_(1,35)_ = 1.20, *p* = 0.28, Figure 2C), suggesting that the observed effects on distance in the center and the number of entries to the center are not solely the result of decreased locomotor activity. Despite the reductions in entries to the center and distance in the center, there was no significant effect of diet on the amount of time spent in the center of the open field (*F*_(1,35)_ = 2.86, *p* = 0.099, Figure 2D). No significant effects of genotype and no genotype by diet interactions were observed.

### Elevated Plus Maze

In the EPM test, KI mice traveled a shorter distance overall than did WT animals (main effect of genotype; *F*_(1,35)_ = 13.09, *p* = 0.0009, Figure 2E). KI mice also traveled a shorter distance in the closed arms of the maze (main effect of genotype; *F*_(1,35)_ = 4.33, *p* = 0.045, Figure 2F) and a shorter distance in the open arms of the maze (main effect of genotype; *F*_(1,35)_ = 9.83, *p* = 0.004, Figure 2G) than did WT. However, there was no significant genotype difference in the amount of time spent in the open arms (*F*_(1,35)_ = 3.13, *p* = 0.086 for effect of genotype, Figure 2H). No significant effects of diet and no genotype by diet interactions were observed.

### Forced Swim Test

For the FST, no significant main effects of diet or genotype were observed. However, there were significant genotype by diet interactions for the number of immobile episodes (*F*_(1,35)_ = 6.91, *p* = 0.013, Figure 2I) and for the time spent immobile (*F*_(1,35)_ = 6.31, *p* = 0.017, Figure 2J). *Post hoc* *t*-tests revealed that HFD led to a significant reduction in immobile episodes in WT mice but had no significant effect in KI animals (Figure 2I). There were no significant genotype differences in FST behavior in mice on SD, but KI mice fed HFD exhibited significantly more immobile episodes and significantly more time spent immobile than did WT mice fed HFD (Figures 2I,J).

### Hippocampal Gene Expression

**Figure 3:**
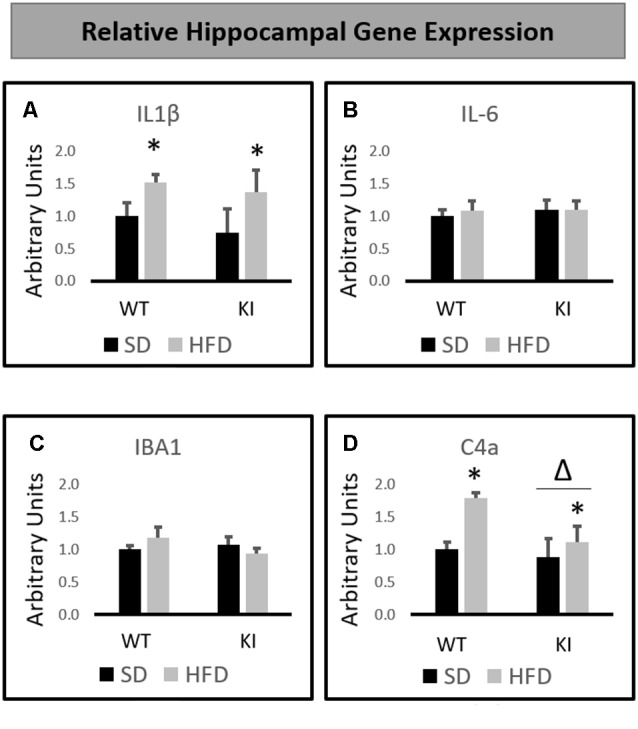
Effects of chronic HFD on WT and KI mice on the expression of inflammation-related genes in the hippocampus. **(A)** Hippocampal expression of interleukin-1β (IL-1β). **(B)** Hippocampal expression of interleukin-6 (IL-6). **(C)** Hippocampal expression of IBA1. **(D)** Hippocampal expression of C4a. All data are normalized to GAPDH. Results are expressed as the mean, and error bars indicate standard error of the mean. “Δ” indicates a main effect of genotype, “*” Indicates a main effect of diet by two-way ANOVA. *N* = 9–11 per group.

HFD significantly increased the hippocampal expression of IL-1β gene (main effect of diet, *F*_(1,32)_ = 5.3, *p* = 0.028, Figure 3A) but had no significant effects of genotype, and no interactions were observed. In contrast, no significant main effects of diet or genotype and no significant interactions were observed for IL-6 (Figure 3B) or IBA1 (Figure 3C) expression. Gene expression analysis revealed that HFD led to a significant increase in the hippocampal expression of the complement 4A (C4A) gene (main effect of diet, *F*_(1,32)_ = 6.99, *p* = 0.013, Figure 3D). In addition, KI mice were observed to exhibit significantly lower levels of C4A than were WT mice (main effect of genotype, *F*_(1,32)_ = 4.33, *p* = 0.046, Figure 3D), but no interactions were observed.

### Hippocampal GSK3β Signaling

**Figure 4:**
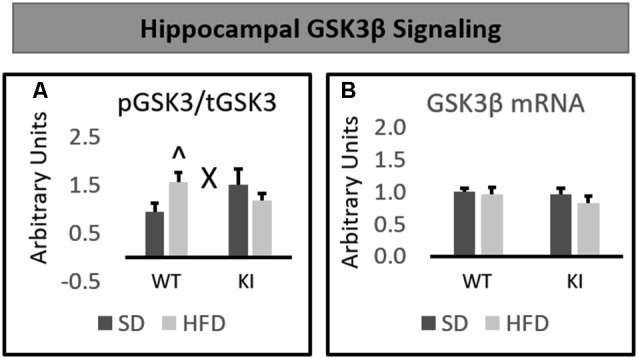
Effects of chronic HFD on WT and KI mice on GSK3β signaling in the hippocampus. **(A)** Quantification of Western blots for phosphorylated GSK3β. Graph shows the ratio of phosphorylated GSK3β to total GSK3β. Representative images are shown below. **(B)** Quantification of mRNA levels of GSK3β normalized to GAPDH. The results are expressed as the mean, and error bars indicate standard error of the mean. “X” Indicates a significant genotype by diet interaction by two-way ANOVA, *p* < 0.05. “∧” Indicates significantly greater than WT-SD by *post hoc* *t*-test. *N* = 8–10 per group.

No significant main effects of genotype or diet were observed on the phosphorylation of GSK3β in the hippocampus. However, a significant genotype by diet interaction was observed on the hippocampal level of phosphorylated GSK3β relative to total GSK3β (*F*_(1,30)_ = 4.74, *p* = 0.0375, Figure 4A). *Post hoc* analyses revealed that HFD led to a significant increase in GSK3β phosphorylation in WT mice, but not Tph2KI animals. No significant alterations in the mRNA levels of GSK3β in the hippocampus were observed (Figure 4B).

## Discussion

The current study shows that HFD led to a significant reduction in immobile episodes in WT mice in the FST, a finding that is consistent with prior studies showing that HFD can lead to therapeutic-like effects (Maniam and Morris, 2010a,b; Finger et al., 2011; Dornellas et al., 2018). The fact that KI animals did not exhibit this response suggests that brain 5-HT may be required for some antidepressant-like effects of HFD. This result is generally consistent with the idea that brain 5-HT plays an important role in antidepressant-like responses, which has also been supported by our previous studies demonstrating impaired fluoxetine responses in Tph2KI mice (Sachs et al., 2013, 2015). Although HFD tended to decrease immobility in WT mice and increase immobility in KI animals, the only HFD-induced alteration in FST behavior that was significant by *post hoc* testing was the reduction in immobile episodes in WT mice. Because HFD-fed KI mice did not display significantly increased immobility than did either genotype of SD-fed animals, it appears that HFD-fed KI mice do not exhibit a “depression-like” state. Rather, they simply fail to experience an antidepressant-like response. Importantly, the validity of the FST as a depression-like behavior remains an area of open debate. Indeed, some have argued that immobility time is a more accurate reflection of an acute stress coping strategy than it is a measure of behavioral despair (Commons et al., 2017). Regardless, 5-HT deficiency-induced alterations in either stress coping or behavioral despair following exposure to HFD could have important implications for mental health.

Although several articles have reported increased immobility in the tail suspension test in KI mice compared with WT (Beaulieu et al., 2008; Dzirasa et al., 2013), we have previously shown that KI animals do not exhibit increased immobility in the FST (Sachs et al., 2014a). The reasons underlying these different effects in these closely related tests remain unclear. However, research using Tph2 knock-out mice, which display even more dramatic reductions in brain 5-HT than Tph2KI mice, has shown that knock-out animals do not exhibit increased immobility in either of these tests (Angoa-Pérez et al., 2014).

The current finding that HFD significantly reduced locomotor activity in the center of the open field and the number of center entries in the OFT, but had no significant effects in the EPM, is similar to prior work by Dutheil et al. (2016) who also reported significantly increased anxiety in the OFT, but not the EPM, in rats exposed to 16 weeks of HFD. Our EPM results did reveal a nearly 40% reduction in open arm time in WT mice following HFD, suggesting that HFD may not have been completely without effect in this test, but this result did not reach statistical significance by two-way ANOVA. Brain 5-HT deficiency led to significant reductions in the distance traveled in all compartments of the EPM, likely reflecting an overall reduction in exploratory drive in this test. However, this may also reflect an increase in anxiety-like behavior in 5-HT-deficient animals, as distance traveled was affected more in the open arms (~55% reduction) than in the center area (~29% reduction) and the closed arms (~17% reduction). In the OFT, brain 5-HT deficiency did not influence the anxiogenic effect of HFD, and no main effects of genotype were observed. Overall, our behavioral results reveal that HFD tends to promote anxiety-like behavior and reduce depression-like behavior in WT mice and that low 5-HT blocks antidepressant-like responses to HFD.

In addition to our behavioral findings, our study also identified genotype differences in GSK3β signaling following HFD. Specifically, HFD increased phosphorylation of GSK3β only in WT mice, not KI animals, and unlike many kinases, phosphorylation of GSK3β is known to inhibit the enzyme. Interestingly, GSK3β inhibition has been shown to lead to antidepressant-like effects in rodents (Gould et al., 2004), including Tph2KI mice (Beaulieu et al., 2008). In addition, GSK3β inhibition has been shown to be required for some responses to antidepressants (Eom and Jope, 2009) and for antidepressant-like responses to ketamine (Beurel et al., 2011). It is important to note that the current data do not directly demonstrate that HFD-induced hippocampal GSK3β inhibition in WT mice is responsible for the antidepressant-like effect observed here. Indeed, whether the GSK3β alterations are related to the behavioral differences would require additional experimentation. Importantly, the effects of 5-HT on GSK3β have been shown to be brain region and context specific. For example, brain 5-HT deficiency has been reported to lead to a baseline reduction in GSK3β phosphorylation in the frontal cortex, but not hippocampus (Beaulieu et al., 2008). Despite the lack of baseline GSK3β phosphorylation changes in the hippocampus, brain 5-HT deficiency has been shown to block the effects of ethanol on GSK3β phosphorylation in this brain region (Sachs et al., 2014b), an effect that is similar to the blockade of HFD-induced increases in GSK3β phosphorylation observed here. Given the importance of brain GSK3β signaling in the context of mental health (Jope and Johnson, 2004; Gould et al., 2006; Shapira et al., 2007; Jope, 2011), future studies should evaluate the combined effects of HFD and 5-HT deficiency in other brain regions and should evaluate the functional role of GSK3β in mediating behavioral consequences of low 5-HT and HFD.

No significant genotype by diet interactions were observed in the expression of inflammation-related genes in the hippocampus. However, HFD was observed to increase IL-1β levels, which is in keeping with previous work (Almeida-Suhett et al., 2017). HFD also increased the expression of complement C4A, which brain 5-HT deficiency was shown to reduce. The complement system is perhaps best known for its role in innate immunity (Ricklin et al., 2016), but it has also been shown to play a major role in synapse elimination (Stevens et al., 2007) and has been implicated in mental illness, most notably schizophrenia (Sekar et al., 2016; Nimgaonkar et al., 2017; Sellgren et al., 2019). Although no genotype by diet interaction was observed for C4A expression, it is possible that the combination of main effects of 5-HT-deficiency and diet on C4A expression could influence complement-mediated pruning and play an important role in the behavioral responses of Tph2KI mice to HFD, but future research would be required to evaluate this. Importantly, mRNA analyses do not represent a completely comprehensive analysis of brain inflammation, and thus, it is still possible that brain 5-HT deficiency impacts inflammatory responses in the brain, but future research would be required to test this possibility.

A limitation of the current study is that it was conducted exclusively in male animals. Given that we have previously reported sex differences in susceptibility to the effects of stress and brain 5-HT deficiency (Sachs et al., 2014a), future research should evaluate the behavioral consequences of chronic HFD in females. Another limitation is that we do not currently know whether fat content itself is driving the HFD-induced molecular and behavioral changes. Whether other types of highly palatable diets, such as those high in sugar, would lead to similar behavioral changes is not known. Similarly, given that mice consumed more HFD than they did SD, it is also possible that increased consumption of SD would lead to similar effects. Future research examining other diets and pair-fed controls will be required to provide insight into these remaining issues.

## Data Availability Statement

The datasets generated for this study are available on request to the corresponding author.

## Ethics Statement

The animal study was reviewed and approved by Villanova University’s IACUC committee.

## Author Contributions

MMK, SG and BS designed the experiments. MMK, MDK, BB, ND, SG and BS performed the experiments. MMK, BB, ND and BS analyzed the data. MMK, MDK, BB and BS wrote the manuscript. All authors approved the final version.

## Conflict of Interest

The authors declare that the research was conducted in the absence of any commercial or financial relationships that could be construed as a potential conflict of interest.

## References

[B1] Abildgaard A. Solskov L. Volke V. Harvey B. H. Lund S. Wegener G. (2011). A high-fat diet exacerbates depressive-like behavior in the Flinders Sensitive Line (FSL) rat, a genetic model of depression. Psychoneuroendocrinology 36, 623–633. 10.1016/j.psyneuen.2010.09.004 20888697

[B2] Almeida-Suhett C. P. Graham A. Chen Y. Deuster P. (2017). Behavioral changes in male mice fed a high-fat diet are associated with IL-1β expression in specific brain regions. Physiol. Behav. 169, 130–140. 10.1016/j.physbeh.2016.11.016 27876639

[B3] Alonso-Caraballo Y. Hodgson K. J. Morgan S. A. Ferrario C. R. Vollbrecht P. J. (2019). Enhanced anxiety-like behavior emerges with weight gain in male and female obesity-susceptible rats. Behav. Brain Res. 360, 81–93. 10.1016/j.bbr.2018.12.002 30521928 PMC6462400

[B4] Angoa-Pérez M. Kane M. J. Briggs D. I. Herrera-Mundo N. Sykes C. E. Francescutti D. M.. (2014). Mice genetically depleted of brain serotonin do not display a depression-like behavioral phenotype. ACS Chem. Neurosci. 5, 908–919. 10.1021/cn500096g 25089765 PMC4777283

[B5] Aslani S. Vieira N. Marques F. Costa P. S. Sousa N. Palha J. A. (2015). The effect of high-fat diet on rat’s mood, feeding behavior and response to stress. Transl. Psychiatry 5:e684. 10.1038/tp.2015.178 26795748 PMC5545690

[B6] Beaulieu J. M. Zhang X. Rodriguiz R. M. Sotnikova T. D. Cools M. J. Wetsel W. C.. (2008). Role of GSK3 β in behavioral abnormalities induced by serotonin deficiency. Proc. Natl. Acad. Sci. U S A 105, 1333–1338. 10.1073/pnas.0711496105 18212115 PMC2234138

[B7] Beurel E. Song L. Jope R. S. (2011). Inhibition of glycogen synthase kinase-3 is necessary for the rapid antidepressant effect of ketamine in mice. Mol. Psychiatry 16, 1068–1070. 10.1038/mp.2011.47 21502951 PMC3200424

[B8] Bridgewater L. C. Zhang C. Wu Y. Hu W. Zhang Q. Wang J.. (2017). Gender-based differences in host behavior and gut microbiota composition in response to high fat diet and stress in a mouse model. Sci. Rep. 7:10776. 10.1038/s41598-017-11069-4 28883460 PMC5589737

[B9] Chen J. Wang M. Waheed Khan R. A. He K. Wang Q. Li Z.. (2015). The GSK3B gene confers risk for both major depressive disorder and schizophrenia in the Han Chinese population. J. Affect. Disord. 185, 149–155. 10.1016/j.jad.2015.06.040 26186530

[B10] Commons K. G. Cholanians A. B. Babb J. A. Ehlinger D. G. (2017). The rodent forced swim test measures stress-coping strategy, not depression-like behavior. ACS Chem. Neurosci. 8, 955–960. 10.1021/acschemneuro.7b00042 28287253 PMC5518600

[B11] Crane J. D. Palanivel R. Mottillo E. P. Bujak A. L. Wang H. Ford R. J.. (2015). Inhibiting peripheral serotonin synthesis reduces obesity and metabolic dysfunction by promoting brown adipose tissue thermogenesis. Nat. Med. 21, 166–172. 10.1038/nm.3766 25485911 PMC5647161

[B12] Dornellas A. P. S. Boldarine V. T. Pedroso A. P. Carvalho L. O. T. de Andrade I. S. Vulcani-Freitas T. M.. (2018). High-fat feeding improves anxiety-type behavior induced by ovariectomy in rats. Front. Neurosci. 12:557. 10.3389/fnins.2018.00557 30233288 PMC6129615

[B13] Dutheil S. Ota K. T. Wohleb E. S. Rasmussen K. Duman R. S. (2016). High-fat diet induced anxiety and anhedonia: impact on brain homeostasis and inflammation. Neuropsychopharmacology 41, 1874–1887. 10.1038/npp.2015.357 26658303 PMC4869056

[B14] Dzirasa K. Kumar S. Sachs B. D. Caron M. G. Nicolelis M. A. (2013). Cortical-amygdalar circuit dysfunction in a genetic mouse model of serotonin deficiency. J. Neurosci. 33, 4505–4513. 10.1523/jneurosci.4891-12.2013 23467366 PMC3633471

[B15] Eom T. Y. Jope R. S. (2009). Blocked inhibitory serine-phosphorylation of glycogen synthase kinase-3α/β impairs *in vivo* neural precursor cell proliferation. Biol. Psychiatry 66, 494–502. 10.1016/j.biopsych.2009.04.015 19520363 PMC2746934

[B16] Faith M. S. Matz P. E. Jorge M. A. (2002). Obesity-depression associations in the population. J. Psychosom. Res. 53, 935–942. 10.1016/s0022-3999(02)00308-2 12377306

[B17] Ferrari A. J. Norman R. E. Freedman G. Baxter A. J. Pirkis J. E. Harris M. G.. (2014). The burden attributable to mental and substance use disorders as risk factors for suicide: findings from the Global Burden of Disease Study 2010. PLoS One 9:e91936. 10.1371/journal.pone.0091936 24694747 PMC3973668

[B18] Finger B. C. Dinan T. G. Cryan J. F. (2011). High-fat diet selectively protects against the effects of chronic social stress in the mouse. Neuroscience 192, 351–360. 10.1016/j.neuroscience.2011.06.072 21742017

[B19] Gariepy G. Nitka D. Schmitz N. (2010). The association between obesity and anxiety disorders in the population: a systematic review and meta-analysis. Int. J. Obes. 34, 407–419. 10.1038/ijo.2009.252 19997072

[B20] Giridharan V. V. Sayana P. Pinjari O. F. Ahmad N. da Rosa M. I. Quevedo J.. (2019). Postmortem evidence of brain inflammatory markers in bipolar disorder: a systematic review. Mol. Psychiatry [Epub ahead of print]. 10.1038/s41380-019-0448-7 31249382

[B21] Gould T. D. Einat H. Bhat R. Manji H. K. (2004). AR-A014418, a selective GSK-3 inhibitor, produces antidepressant-like effects in the forced swim test. Int. J. Neuropsychopharmacol. 7, 387–390. 10.1017/s1461145704004535 15315719

[B22] Gould T. D. Picchini A. M. Einat H. Manji H. K. (2006). Targeting glycogen synthase kinase-3 in the CNS: implications for the development of new treatments for mood disorders. Curr. Drug Targets 7, 1399–1409. 10.2174/1389450110607011399 17100580

[B23] Jacobsen J. P. Siesser W. B. Sachs B. D. Peterson S. Cools M. J. Setola V.. (2012). Deficient serotonin neurotransmission and depression-like serotonin biomarker alterations in tryptophan hydroxylase 2 (Tph2) loss-of-function mice. Mol. Psychiatry 17, 694–704. 10.1038/mp.2011.50 21537332 PMC3536482

[B24] Jope R. S. (2011). Glycogen synthase kinase-3 in the etiology and treatment of mood disorders. Front. Mol. Neurosci. 4:16. 10.3389/fnmol.2011.00016 21886606 PMC3152743

[B25] Jope R. S. Johnson G. V. (2004). The glamour and gloom of glycogen synthase kinase-3. Trends Biochem. Sci. 29, 95–102. 10.1016/j.tibs.2003.12.004 15102436

[B26] Karege F. Perroud N. Burkhardt S. Fernandez R. Ballmann E. La Harpe R.. (2012). Protein levels of β-catenin and activation state of glycogen synthase kinase-3β in major depression. A study with postmortem prefrontal cortex. J. Affect. Disord. 136, 185–188. 10.1016/j.jad.2011.09.024 22036797

[B27] Kessler R. C. Avenevoli S. Costello E. J. Georgiades K. Green J. G. Gruber M. J.. (2012). Prevalence, persistence and sociodemographic correlates of DSM-IV disorders in the National Comorbidity Survey Replication Adolescent Supplement. Arch. Gen. Psychiatry 69, 372–380. 10.1001/archgenpsychiatry.2011.160 22147808 PMC3445020

[B28] Khodanovich M. Kisel A. Kudabaeva M. Chernysheva G. Smolyakova V. Krutenkova E.. (2018). Effects of fluoxetine on hippocampal neurogenesis and neuroprotection in the model of global cerebral ischemia in rats. Int. J. Mol. Sci. 19:E162. 10.3390/ijms19010162 29304004 PMC5796111

[B29] Kurhe Y. Mahesh R. Devadoss T. (2017). Novel 5-HT_3_ receptor antagonist QCM-4 attenuates depressive-like phenotype associated with obesity in high-fat-diet-fed mice. Psychopharmacology 234, 1165–1179. 10.1007/s00213-017-4558-0 28238069

[B31] Li Y. Lv M. R. Wei Y. J. Sun L. Zhang J. X. Zhang H. G.. (2017). Dietary patterns and depression risk: a meta-analysis. Psychiatry Res. 253, 373–382. 10.1016/j.psychres.2017.04.020 28431261

[B30] Li X. Zhu W. Roh M. S. Friedman A. B. Rosborough K. Jope R. S. (2004). *In vivo* regulation of glycogen synthase kinase-3β (GSK3β) by serotonergic activity in mouse brain. Neuropsychopharmacology 29, 1426–1431. 10.1038/sj.npp.1300439 15039769 PMC1986663

[B32] Lu Y. Ho C. S. Liu X. Chua A. N. Wang W. McIntyre R. S.. (2017). Chronic administration of fluoxetine and pro-inflammatory cytokine change in a rat model of depression. PLoS One 12:e0186700. 10.1371/journal.pone.0186700 29049348 PMC5648231

[B33] Luppino F. S. de Wit L. M. Bouvy P. F. Stijnen T. Cuijpers P. Penninx B. W.. (2010). Overweight, obesity, and depression: a systematic review and meta-analysis of longitudinal studies. Arch. Gen. Psychiatry 67, 220–229. 10.1001/archgenpsychiatry.2010.2 20194822

[B34] Maniam J. Morris M. J. (2010a). Long-term postpartum anxiety and depression-like behavior in mother rats subjected to maternal separation are ameliorated by palatable high fat diet. Behav. Brain Res. 208, 72–79. 10.1016/j.bbr.2009.11.005 19896506

[B35] Maniam J. Morris M. J. (2010b). Voluntary exercise and palatable high-fat diet both improve behavioural profile and stress responses in male rats exposed to early life stress: role of hippocampus. Psychoneuroendocrinology 35, 1553–1564. 10.1016/j.psyneuen.2010.05.012 20594764

[B36] Nimgaonkar V. L. Prasad K. M. Chowdari K. V. Severance E. G. Yolken R. H. (2017). The complement system: a gateway to gene-environment interactions in schizophrenia pathogenesis. Mol. Psychiatry 22, 1554–1561. 10.1038/mp.2017.151 28761078 PMC5656502

[B37] Ogrodnik M. Zhu Y. Langhi L. G. P. Tchkonia T. Kruger P. Fielder E.. (2019). Obesity-induced cellular senescence drives anxiety and impairs neurogenesis. Cell Metab. 29:1233. 10.1016/j.cmet.2019.01.013 31067450 PMC6509279

[B38] Opel N. Cearns M. Clark S. Toben C. Grotegerd D. Heindel W.. (2019). Large-scale evidence for an association between low-grade peripheral inflammation and brain structural alterations in major depression in the BiDirect study. J. Psychiatry Neurosci. 44, 423–431. 10.1503/jpn.180208 31304733 PMC6821515

[B39] Osimo E. F. Baxter L. J. Lewis G. Jones P. B. Khandaker G. M. (2019). Prevalence of low-grade inflammation in depression: a systematic review and meta-analysis of CRP levels. Psychol. Med. 2019, 1–13. 10.1017/s0033291719001454 31258105 PMC6712955

[B40] Pan Q. Liu Q. Wan R. Kalavagunta P. K. Liu L. Lv W.. (2019). Selective inhibition of intestinal 5-HT improves neurobehavioral abnormalities caused by high-fat diet mice. Metab. Brain Dis. 34, 747–761. 10.1007/s11011-019-0392-x 30931486

[B41] Papazoglou I. K. Jean A. Gertler A. Taouis M. Vacher C. M. (2015). Hippocampal GSK3β as a molecular link between obesity and depression. Mol. Neurobiol. 52, 363–374. 10.1007/s12035-014-8863-x 25169083

[B42] Rebolledo-Solleiro D. Roldán-Roldán G. Díaz D. Velasco M. Larqué C. Rico-Rosillo G.. (2017). Increased anxiety-like behavior is associated with the metabolic syndrome in non-stressed rats. PLoS One 12:e0176554. 10.1371/journal.pone.0176554 28463967 PMC5413000

[B43] Ren X. Rizavi H. S. Khan M. A. Dwivedi Y. Pandey G. N. (2013). Altered Wnt signalling in the teenage suicide brain: focus on glycogen synthase kinase-3β and β-catenin. Int. J. Neuropsychopharmacol. 16, 945–955. 10.1017/s1461145712001010 23110823

[B44] Ricklin D. Reis E. S. Mastellos D. C. Gros P. Lambris J. D. (2016). Complement component C3—the “swiss army knife” of innate immunity and host defense. Immunol. Rev. 274, 33–58. 10.1111/imr.12500 27782325 PMC5427221

[B45] Ronai Z. Kovacs-Nagy R. Szantai E. Elek Z. Sasvari-Szekely M. Faludi G.. (2014). Glycogen synthase kinase 3 β gene structural variants as possible risk factors of bipolar depression. Am. J. Med. Genet. B Neuropsychiatr. Genet. 165B, 217–222. 10.1002/ajmg.b.32223 24677591 PMC3980030

[B46] Sachs B. D. Jacobsen J. P. Thomas T. L. Siesser W. B. Roberts W. L. Caron M. G. (2013). The effects of congenital brain serotonin deficiency on responses to chronic fluoxetine. Transl. Psychiatry 3:e291. 10.1038/tp.2013.65 23942622 PMC3756292

[B47] Sachs B. D. Ni J. R. Caron M. G. (2014a). Sex differences in response to chronic mild stress and congenital serotonin deficiency. Psychoneuroendocrinology 40, 123–129. 10.1016/j.psyneuen.2013.11.008 24485484 PMC3918518

[B49] Sachs B. D. Salahi A. A. Caron M. G. (2014b). Congenital brain serotonin deficiency leads to reduced ethanol sensitivity and increased ethanol consumption in mice. Neuropharmacology 77C, 177–184. 10.1016/j.neuropharm.2013.09.010 24067926 PMC3874885

[B48] Sachs B. D. Ni J. R. Caron M. G. (2015). Brain 5-HT deficiency increases stress vulnerability and impairs antidepressant responses following psychosocial stress. Proc. Natl. Acad. Sci. U S A 112, 2557–2562. 10.1073/pnas.1416866112 25675490 PMC4345581

[B50] Sachs B. D. Tran H. L. Folse E. Caron M. G. (2018). Brain-region-specific molecular responses to maternal separation and social defeat stress in mice. Neuroscience 373, 122–136. 10.1016/j.neuroscience.2018.01.018 29341883 PMC5816704

[B51] Sekar A. Bialas A. R. de Rivera H. Davis A. Hammond T. R. Kamitaki N.. (2016). Schizophrenia risk from complex variation of complement component 4. Nature 530, 177–183. 10.1038/nature16549 26814963 PMC4752392

[B52] Sellgren C. M. Gracias J. Watmuff B. Biag J. D. Thanos J. M. Whittredge P. B.. (2019). Increased synapse elimination by microglia in schizophrenia patient-derived models of synaptic pruning. Nat. Neurosci. 22, 374–385. 10.1038/s41593-018-0334-7 30718903 PMC6410571

[B53] Shapira M. Licht A. Milman A. Pick C. G. Shohami E. Eldar-Finkelman H. (2007). Role of glycogen synthase kinase-3β in early depressive behavior induced by mild traumatic brain injury. Mol. Cell. Neurosci. 34, 571–577. 10.1016/j.mcn.2006.12.006 17289399

[B54] Sharma S. Fulton S. (2013). Diet-induced obesity promotes depressive-like behaviour that is associated with neural adaptations in brain reward circuitry. Int. J. Obes. 37, 382–389. 10.1038/ijo.2012.48 22508336

[B55] Stevens B. Allen N. J. Vazquez L. E. Howell G. R. Christopherson K. S. Nouri N.. (2007). The classical complement cascade mediates CNS synapse elimination. Cell 131, 1164–1178. 10.1016/j.cell.2007.10.036 18083105

[B56] Strekalova T. Evans M. Costa-Nunes J. Bachurin S. Yeritsyan N. Couch Y.. (2015). Tlr4 upregulation in the brain accompanies depression- and anxiety-like behaviors induced by a high-cholesterol diet. Brain Behav. Immun. 48, 42–47. 10.1016/j.bbi.2015.02.015 25712260

[B57] Stunkard A. J. Faith M. S. Allison K. C. (2003). Depression and obesity. Biol. Psychiatry 54, 330–337. 10.1016/s0006-3223(03)00608-5 12893108

[B58] Sullivan E. L. Grayson B. Takahashi D. Robertson N. Maier A. Bethea C. L.. (2010). Chronic consumption of a high-fat diet during pregnancy causes perturbations in the serotonergic system and increased anxiety-like behavior in nonhuman primate offspring. J. Neurosci. 30, 3826–3830. 10.1523/JNEUROSCI.5560-09.2010 20220017 PMC2846411

[B59] Syed S. A. Beurel E. Loewenstein D. A. Lowell J. A. Craighead W. E. Dunlop B. W.. (2018). Defective inflammatory pathways in never-treated depressed patients are associated with poor treatment response. Neuron 99, 914.e3–924.e3. 10.1016/j.neuron.2018.08.001 30146307 PMC6151182

[B60] Wakabayashi C. Kunugi H. (2019). Involvement of IL-6 and GSK3β in impaired sensorimotor gating induced by high-fat diet. Neurosci. Res. 147, 33–38. 10.1016/j.neures.2018.10.004 30326250

[B61] Wang X. Spandidos A. Wang H. Seed B. (2012). PrimerBank: a PCR primer database for quantitative gene expression analysis, 2012 update. Nucleic Acids Res. 40, D1144–D1149. 10.1093/nar/gkr1013 22086960 PMC3245149

[B62] Wu H. Liu Q. Kalavagunta P. K. Huang Q. Lv W. An X.. (2018). Normal diet Vs. High fat diet—a comparative study: behavioral and neuroimmunological changes in adolescent male mice. Metab. Brain Dis. 33, 177–190. 10.1007/s11011-017-0140-z 29101600

[B63] Xu L. Xu S. Lin L. Gu X. Fu C. Fang Y.. (2018). High-fat diet mediates anxiolytic-like behaviors in a time-dependent manner through the regulation of SIRT1 in the brain. Neuroscience 372, 237–245. 10.1016/j.neuroscience.2018.01.001 29331532

